# *Bradyrhizobium ottawaense* efficiently reduces nitrous oxide through high* nosZ* gene expression

**DOI:** 10.1038/s41598-023-46019-w

**Published:** 2023-11-01

**Authors:** Sawa Wasai-Hara, Manabu Itakura, Arthur Fernandes Siqueira, Daisaku Takemoto, Masayuki Sugawara, Hisayuki Mitsui, Shusei Sato, Noritoshi Inagaki, Toshimasa Yamazaki, Haruko Imaizumi-Anraku, Yoshikazu Shimoda, Kiwamu Minamisawa

**Affiliations:** 1 Institute of Agrobiological Sciences, National Agriculture and Food Research Organization (NARO), Tsukuba, Ibaraki Japan; 2 Graduate School of Life Sciences, Tohoku University, Sendai, Miyagi Japan; 3 Research Center for Advanced Analysis, National Agriculture and Food Research Organization (NARO), Tsukuba, Ibaraki Japan

**Keywords:** Applied microbiology, Bacterial physiology, Bacterial techniques and applications, Soil microbiology

## Abstract

N_2_O is an important greenhouse gas influencing global warming, and agricultural land is the predominant (anthropogenic) source of N_2_O emissions. Here, we report the high N_2_O-reducing activity of *Bradyrhizobium ottawaense*, suggesting the potential for efficiently mitigating N_2_O emission from agricultural lands. Among the 15 *B. ottawaense* isolates examined, the N_2_O-reducing activities of most (13) strains were approximately five-fold higher than that of *Bradyrhizobium diazoefficiens* USDA110^T^ under anaerobic conditions. This robust N_2_O-reducing activity of *B. ottawaense* was confirmed by N_2_O reductase (NosZ) protein levels and by mitigation of N_2_O emitted by nodule decomposition in laboratory system. While the NosZ of *B. ottawaense* and *B. diazoefficiens* showed high homology, *nosZ* gene expression in *B*. *ottawaense* was over 150-fold higher than that in *B. diazoefficiens* USDA110^T^, suggesting the high N_2_O-reducing activity of *B. ottawaense* is achieved by high *nos* expression. Furthermore, we examined the *nos* operon transcription start sites and found that, unlike *B. diazoefficiens*, *B*. *ottawaense* has two transcription start sites under N_2_O-respiring conditions, which may contribute to the high *nosZ* expression*.* Our study indicates the potential of *B. ottawaense* for effective N_2_O reduction and unique regulation of *nos* gene expression towards the high performance of N_2_O mitigation in the soil.

## Introduction

The expansion of human activities is triggering irreversible environmental damage, including global warming and stratospheric ozone depletion. N_2_O is a long-lived greenhouse gas (GHG) whose atmospheric lifetime is an estimated 116 ± 9 years^1^. Moreover, N_2_O has a stratospheric ozone-depleting effect. Although N_2_O concentration in the atmosphere is still low compared with other GHG such as CO_2_ and CH_4_, N_2_O is an alarming GHG due to its high global warming potential per unit^2^. Agricultural land is the primary source of N_2_O, accounting for 52% of anthropogenic origin emissions^3^. N_2_O is markedly emitted from nitrogen-rich environments, such as agricultural fields in which excess N fertilizers are applied and crop residues, including nodulated legume roots^4,5^. Biochemically, microbial nitrification and denitrification are the two major processes of N_2_O generation^6,7^. During nitrification, N_2_O is produced as a byproduct when ammonia is oxidized to nitrite via hydroxylamine. N_2_O is also generated from NO during incomplete denitrification, which intricately involves diverse soil bacteria, fungi, and archaea^8^. However, to date, only one microbial enzyme, N_2_O reductase (encoded by the *nosZ* gene), reportedly reduces N_2_O to N_2_^7^.

Since some rhizobial species possess the *nosZ* gene, strategies to reduce N_2_O emissions from agricultural fields using rhizobia have been studied. In particular, soybeans are grown globally, and the amount of N_2_O emitted from nodulated soybeans is higher than that from corn or wheat. For example, N_2_O emissions from soybean fields in Argentina are estimated to reach 5.1 kg N ha^−1^ year^−1^^9^. The use of rhizobia is, therefore, an effective approach to reducing global GHG emissions. *Bradyrhizobium* nodulates various legumes, including soybean, and has been studied as a model denitrification microorganism. Soybean roots nodulated with *Bradyrhizobium diazoefficiens* USDA110^T^ scavenges exogenous N_2_O, even in ambient air containing a low concentration of N_2_O (0.34 ppm)^10^. Moreover, N_2_O fluxes from soybean fields have been mitigated by inoculation with *B. diazoefficiens* mutants with high N_2_O reductase activity (*nos*^++^ mutants)^11^. The utility of *B. diazoefficiens* in N_2_O mitigation has also been verified in soybean ecosystems in Japan^12^, France^13^, and South America^14^.

On the other hand, rhizobial strains carrying *nos* genes are uncommon; *nos* genes and N_2_O-reducing activity have been observed only in *B. diazoefficiens*, soybean rhizobia^10^, and *Ensifer meliloti*, an alfalfa endosymbiont^15^. Several soybean rhizobia species, including *B. diazoefficiens*, *B*. *japonicum*, *B. elkanii*, and *Ensifer fredii,* have been identified, but most soybean rhizobia in Japan and the world are non-*nos*-possessing (*nos*−) species^16^. However, *nos* gene clusters have been recently found in *Bradyrhizobium ottawaense*^17^ and *Rhizobium leguminosarum*^13^, suggesting that the strategy for mitigating N_2_O emissions from the legume rhizosphere using rhizobia could be expanded to various legume and rhizobial species.

Soybean rhizosphere is a hotspot for complicated nitrogen transformations including production and reduction of N_2_O. Nodule decomposition is a major source of N_2_O through ammonification, nitrification and denitrification in soil organisms and rhizobia^18^. N_2_O is only emitted by decomposed nodules at the late growth period of soybean growth, but not by fresh nodules or roots^5,11,19–21^. N_2_O formed by nodule decomposition is either emitted into the atmosphere or is further reduced to N_2_ by N_2_O reductase of soybean bradyrhizobia possessing *nosZ* gene (*nos*^+^ strains)^10,21,22^. Due to the balance between the production and reduction of N_2_O during nodule decomposition, N_2_O emission occurs in root systems with nodules harboring *nos*^–^ and even *nos*^+^ strains. Therefore, to effectively prevent N_2_O release, using rhizobia with high N_2_O-reducing activity is necessary.

Denitrification reactions involving N_2_O reduction occur under anaerobic conditions. In bradyrhizobia, N_2_O reductase (*nos*) genes are regulated by three different two-component regulatory systems^18^. The FixLJK_2_ cascade is the primary oxygen-sensing regulator for *nos* operons. Under moderate low oxygen concentration conditions (< 5%), FixLJK_2_ recognizes the FixK box [TTG(A/C)-N_6_-(T/G)CAA] located upstream of *nosR* and promotes *nos* operon expression^23,24^. It has also been shown that the NasST two-component regulatory system, which senses NO_3_^-^ concentrations and regulates the NO_3_^-^ assimilation gene (*nas*) operon, is also responsible for regulating the *nos* operon^25^. NasT act as activators of the *nas*/*nos* operons and NasS acts on NasT, inhibiting its function: in the absence of NO_3_^-^/NO_2_^-^, NasS and NasT bind to each other, and transcription is arrested by the terminator structure upstream of the *nas*/*nos* operon. On the other hand, in the presence of NO_3_^-^/NO_2_^-^, NasT is released from NasS and binds to the mRNA upstream of the *nos* operon, resulting in a conformational change in the hairpin termination structure of the mRNA and read-through transcription of the *nos* genes^18^. In *nasS* deletion mutants, transcription of the *nos* operon is activated independently of NO_3_^-^. Itakura et al.^11^ developed *nos*^++^ strains from naturally occurring *nasS* mutants and verified their utility in N_2_O reduction in laboratory and field experiments. Additionally, the RegSR two-component regulatory system presumably controls *nosR* expression via the NifA protein^26^.

The catalytic unit of N_2_O reductase requires auxiliary proteins which are encoded in *nos* gene cluster (*nosRZDFYLX*)^27^. The flavoproteins NosR and NosX form an electron transport pathway from the quinone pool to NosZ. NosR is also required for the transcription of nos genes. NosD, NosF, NosY, and NosL are involved in maturation of the CuZ site of NosZ^27,28^.

In this study, we characterized *nos*-possessing *B. ottawaense* strains isolated from sorghum roots based on their genome sequence and activity. *B. ottawaense* has been reported to form effective nitrogen fixing nodules on soybeans^29^ and nitrogen fixation activity was comparable to that of *B. diazoefficiens* at the plant level^30^. Most *B. ottawaense* strains analyzed in this study showed significantly higher N_2_O-reducing activity than that of *B. diazoefficiens* USDA110^T^. Gene expression and promoter analyses showed that *B. ottawaense* strongly expressed the *nosZ* gene under both N_2_O- and NO_3_^-^-reducing conditions, and its high-level expression is thought to be achieved by different *nos* operon transcription start sites and not by already known regulation systems. Our study indicates the potential of *B. ottawaense* in N_2_O mitigation and the unique regulation of *nos* gene expression that contributes to the high performance of N_2_O reduction.

## Results

### ***N***_***2***_***O- ***reducing activity in *B. ottawaense*

The *B. ottawaense* strains used in this study are listed in Supplementary Table 1. Among them, the phylogenetic relationships and gene conservation of the denitrification pathway of four strains (SG09, TM102, TM233, and TM239) have been reported^17^. To confirm species classification and gene organization, we determined the draft genome sequence of 10 strains, including 3 strains reported by Wasai-Hara et al.^17^ (see Supplementary Table 1). All isolates showed more than 95.0% average nucleotide identity (ANI) values with the type strain *B. ottawaense* OO99^T^^31^, indicating that the isolates were classified into *B*. *ottawaense* (see Supplementary Table 2). Furthermore, phylogenetic analysis based on multiple housekeeping genes (AMPHORA^32^) supported this classification, as shown in Supplementary Fig. 1.

**Figure 1:**
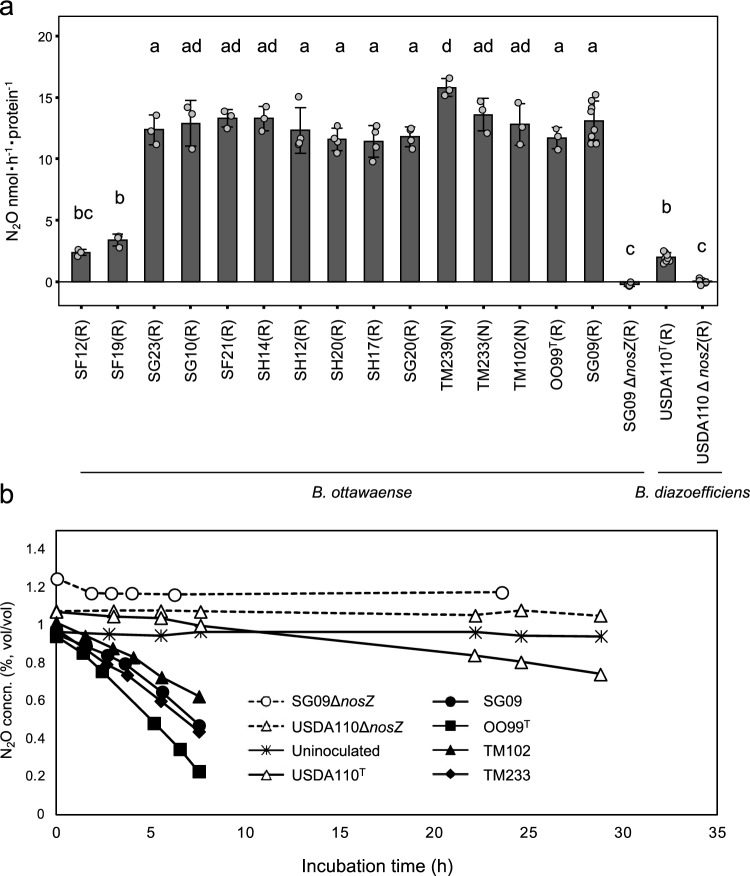
N_2_O-reducing activity of *B. ottawaense.* (**a**) N_2_O-reducing activity of *B. ottawaense* isolates, type strain OO99^T^, *B. diazoefficiens* stain USDA110^T^, and the *nosZ*-deficient strain (Δ*nosZ*). Different letters above the bars represent significant differences between inoculation treatments analyzed using Tukey’s test after analysis of variance (ANOVA; *p* < 0.05). Parentheses after the strain name indicate nodule-forming ability. R = nodule forming strain (rhizobia), N = non-nodulation and non-diazotroph. (**b**) N_2_O-reducing activity in representative strains of *B. ottawaense* and *B. diazoefficiens*. The graph shows the changes in N_2_O concentration over time in the gas phase in the test tube.

The N_2_O-reducing activity of the *B. ottawaense* strains was determined under free-living, N_2_O-respiring conditions (Fig. 1a, see also Supplementary Fig. 2). Almost all isolates and the type strain *B. ottawaense* OO99^T^ showed activity in the range of 11.5–15.8 nmol h^−1^ protein^−1^, which was 5.5–7.4-fold higher than that of *B. diazoefficiens* USDA110^T^ (2.0 nmol h^−1^ protein^−1^). Also, growth under N_2_O-respiring conditions was better in *B. ottawaense* strains than in *B*. *diazoefficiens* USDA110^T^ (Supplementary Fig. 3). Conversely, two strains (SF12 and SF19) showed relatively low activity, with values of 2.4 and 3.4 nmol h^−1^ protein^−1^, respectively, comparable to that of *B. diazoefficiens* USDA110^T^ (Fig. 1a). We also analyzed the N_2_O-reducing activity of TM102, TM233, and TM239, which lack nodulation and nitrogen-fixing ability on soybeans^17^, but no significant difference was observed from the other nodulating strains of *B. ottawaense* (p < 0.05, Tukey’s test). Monitoring N_2_O concentrations over time showed a rapid decrease in *B. ottawaense* (SG09, OO99^T^, TM102, and TM233), while *B. diazoefficiens* USDA110^T^ exhibited a slow decrease (Fig. 1b).

**Figure 2:**
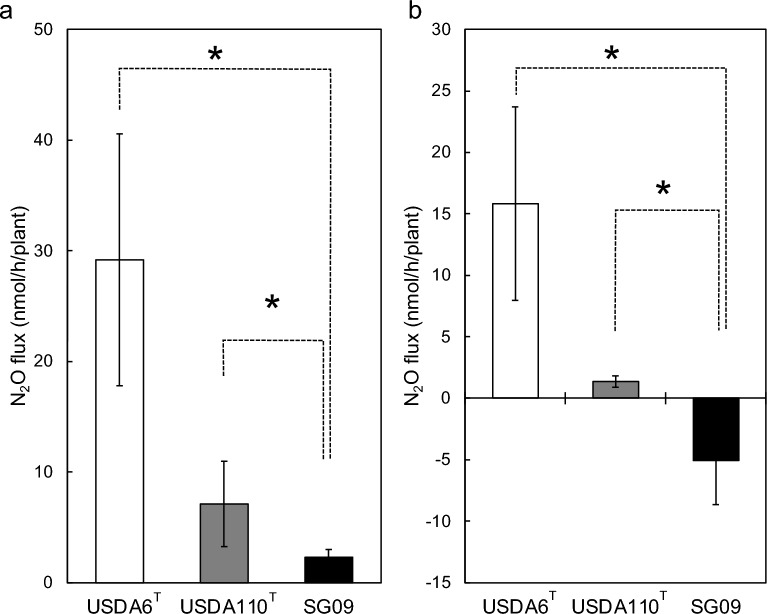
N_2_O flux in rhizosphere inoculated with either *Bradyrhizobium ottawaense*, *B. diazoefficiens* and *B. japonicum.* N_2_O flux from the rhizosphere of soybean plants inoculated with *B. ottawaense* SG09 (*nos* ^+^), *B. diazoefficiens* USDA110^T^ (*nos*^+^), and *B. japonicum* USDA 6^T^ (*nos*^*−*^) under (**a**) an atmospheric concentration (approximately 340 ppb) of N_2_O and (**b**) N_2_O-supplemented air (50 ppm). Asterisks represent significant differences at *p* < 0.05 by the *t*-test.

Next, we examined the effects of *B. ottawaense* inoculation on the N_2_O flux associated with nodule degradation in a soybean rhizosphere in a laboratory system (Fig. 2, Supplementary Fig. 4). After soybean seeds (cv Enrei) were inoculated with *B. japonicum* USDA6^T^ (*nos*^-^), *B. diazoefficiens* USDA110^T^ (*nos*^+^) and *B. ottawaense* SG09 (*nos*^+^), the root system of 36 days-old soybean plants (the nodule fresh weights were similar (1.9–2.1 g fresh weight plant^-1^) irrespective to USDA6^T^, USDA110^T^ and SG09 inoculation treatments) were decapitated and transferred into open bottles containing a field soil. After 20 days incubation, we determined N_2_O flux from the nodulating root-soil system containing decomposing nodules in closed bottles under an aerobic condition, which simulates the rhizosphere of field-grown soybeans. Under atmospheric conditions (approximately 340 ppb of N_2_O in air), N_2_O flux from soybean rhizospheres inoculated with *B. japonicum* USDA 6^T^, *B. diazoefficiens* USDA110^T^ and *B. ottawaense* SG09 was 29.2, 7.1 and 2.3 nmol h^−1^ plant^−1^, respectively (Fig. 2a). N_2_O flux following SG09 inoculation significantly decreased relative to that after *B. japonicum* USDA 6^T^ (*nos*^−^) and *B. diazoefficiens* USDA110^T^ (*nos*^+^) inoculation. N_2_O flux of USDA110^T^ inoculation also significantly decreased relative to that *B. japonicum* USDA 6^T^ (*nos*^−^) that is similar to the previous results^11^. In N_2_O-supplemented air (50 ppm N_2_O), *B. ottawaense* SG09 inoculation exclusively showed negative N_2_O flux. However, such negative flux was not observed with *B. diazoefficiens* USDA 110^T^ and USDA 6^T^ inoculation (Fig. 2b). These results demonstrate the effectiveness of *B. ottawaense* inoculation in reducing N_2_O emissions from the soybean rhizosphere.

### *nosZ* gene expression and protein activity of wild-type *B. ottawaense* strains

*nosZ* expression in *B. ottawaense* strains was evaluated by RT-qPCR under both N_2_O- and NO_3_^-^-respiring conditions based on *B. diazoefficiens* USDA110^T^ (Table 1). Under N_2_O-respiring conditions, wild-type (WT) *B. ottawaense* SG09 and OO99^T^ strains showed 211.5- and 163.5-fold higher expression levels than WT *B. diazoefficiens* USDA110^T^, respectively. Under NO_3_^-^-respiring conditions, the *nosZ* expression of WT strains was upregulated in both *B*. *ottawaense* and *B*. *diazoefficiens* being 19.3-fold (USDA110^T^), 8.9-fold (SG09), and 12.6-fold (OO99^T^) higher than that under N_2_O-respiring conditions. In the comparison among strains, *B*. *ottawaense* SG09 and OO99^T^ showed 109- and 119-fold higher *nosZ* expression than that of USDA110^T^ respectively, even under NO_3_^-^-respiring conditions. On the other hand, the two strains with low N_2_O-reducing activity (SF12 and SF19; Fig. 1a) showed low expression levels that were 31.2- and 40.6-fold higher than those of USDA110^T^, respectively, and less than 1/5 those of SG09 under N_2_O-respiring conditions.

**Table 1:** Relative expression of *nosZ* under N_2_O- and NO_3_^-^-respiring conditions.

	N_2_O-respiring condition	NO_3_^−^-respiring condition	Ratio of *nosZ* expression of NO_3_^-^/N_2_O-respiration
*B. diazoefficiens* USDA110^T^	1.0 ± 0.48	19.3 (1.0^†^) ± 11.4	19.3**
*B. ottawaense* SG09	211.5* ± 58	1880* (109^†^) ± 1090	8.9**
*B. ottawaense* OO99^T^	163.5* ± 81	2064* (119^†^) ± 1110	12.6**
*B. ottawaense* SF12	31.2 ± 8.3	nm	
*B. ottawaense* SF19	40.6 ± 8.2	nm	

Expression is shown relative to *B. diazoefficiens* USDA110^T^ under N_2_O-respiring condition that is set to 1.0 and normalized by *sigA* gene expression. Numbers represent the mean values with standard error of more than three independent experiments.

nm , not measured.

^†^The numbers in parentheses are the relative to USDA110^T^ under NO_3_^-^-respiring condition that is set to 1.0.

*Significant difference between USDA110^T^ and *B. ottawaense* strains at *p* < 0.05, n = 4–6, by *t*-test.

**Significant difference between N_2_O- and NO_3_^-^- respiring conditions at *p* < 0.05, n = 4–6, by *t*-test.

**Figure 3:**
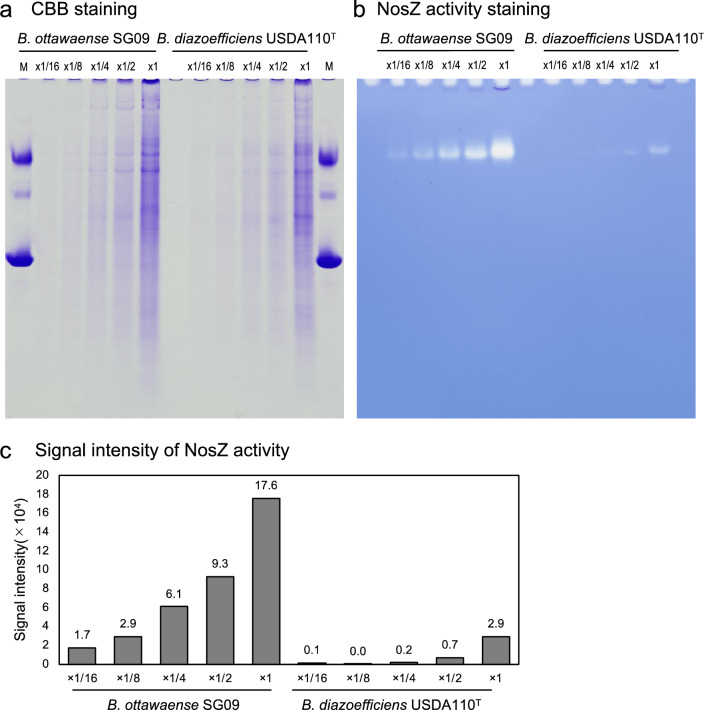
Activity of the NosZ protein of *Bradyrhizobium ottawaense* and *B. diazoefficiens*. Coomassie brilliant blue staining (**a**) and NosZ-specific activity staining (DOC-PAGE, **b**) protein extracted from *B. ottawaense* SG09 and *B. diazoefficiens* USDA110^T^. The numbers in each lane indicate the concentration (*x*) rate of extracted protein samples. ‘M’ indicates the protein size marker (60, 120, and 240 kDa were indicated). The gel images are cropped; full images are shown in Supplemental Fig. 5. Panel (**c**) shows the signal intensity of the NosZ-specific activity staining.

We next analyzed NosZ protein activity in *B. ottawaense* and *B. diazoefficiens* by specific activity staining with methyl viologen after sodium deoxycholate polyacrylamide gel electrophoresis (DOC-PAGE). Equal amounts of *B. ottawaense* and *B. diazoefficiens* total protein were loaded and confirmed by Coomassie brilliant blue (CBB) staining (Fig. 3a, see also Supplementary Fig. 5). On the same gel, the intensity of activity staining was clearly higher for SG09 than that for USDA110^T^; the intensity of the eightfold diluted SG09 lane was comparable to that of the non-diluted USDA110^T^ lane, indicating that NosZ protein activity in *B. ottawaense* was approximately eightfold higher than that in *B. diazoefficiens* (Fig. 3b, c).

### *nosZ* expression in *nasS* deletion mutants

To investigate the high *nosZ* expression in *B. ottawaense*, we compared the sequences of the genes involved in the expression of the *nos* operon, *nasST*, *fixLJK*_2_, and *regSR*, in *B. ottawaense* SG09 and *B. diazoefficiens* USDA110^T^. As shown in Supplementary Table 3, all genes showed > 90% identity in amino acid sequence. However, the upstream sequence of the *nos* operon, which is recognized by NasT to suppress transcription, showed only 48% identity in the nucleotide sequence. Therefore, we examined whether the NasST regulatory system is also functional in *B. ottawaense*, similar to *B. diazoefficiens* USDA110^T^^25^. To this end, we analyzed *nosZ* gene expression in *nasS* deletion mutants of *B. ottawaense* SG09 and OO99^T^ (Table 2). In *B*. *diazoefficiens* USDA110^T^, *nosZ* expression was significantly increased (3.4-fold) in the Δ*nasS* mutant. Similarly, *nosZ* expression was significantly increased in the *B. ottawaense* SG09 (2.03-fold) and OO99^T^ (2.06-fold) Δ*nasS* mutants under N_2_O-respiring conditions. This significant expression increase in *nasS* deletion mutants was not observed in the presence of NO_3_^-^. The effect of *nasS* deletion was also observed in N_2_O-reducing activity, although the N_2_O-reducing activities of Δ*nasS* mutants of *B. ottawaense* SG09 and OO99^T^ were not significantly higher than those of their parent strains SG09 and OO99^T^, respectively (See Supplementary Fig. 6).

**Table 2:** Relative expression of *nosZ* in Δ*nasS* strains under N_2_O- and NO_3 _^- ^-respiring conditions.

	N_2_O-respiring condition			NO_3_^-^-respiring condition		
	Wild type^†^	Δ*nasS*	*nasS* deletion effects	Wild type^†^	Δ*nasS*	*nasS* deletion effects
*B. diazoefficiens* USDA110^T^	1.0 ± 0.48	3.4 ± 1.7	3.4*	1.0 ± 0.7	1.1 ± 10.3	1.1 (ns)
*B. ottawaense* SG09	211.5 ± 58	430.2 ± 164	2.03*	109 ± 63	89.3 ± 42.8	0.82 (ns)
*B. ottawaense* OO99^T^	163.5 ± 81	337.4 ± 132	2.06*	119 ± 64	141 ± 53	1.18 (ns)

Expression is shown relative to *B. diazoefficiens* USDA110^T^ that is set to 1.0 and normalized by *sigA* gene expression.

Numbers represent the mean values with standard error of more than three independent experiments.

ns, not significant.

^†^The data for wild type are taken from Table 1.

*Significant difference between wild type and Δ*nasS* strains at *p* < 0.05, n = 4–6, by *t*-test.

### *Nos* operon transcription system in *B. ottawaense*

To investigate the effect of the transcriptional regulation of the *nos* operon on *nosZ* expression, we determined the transcriptional start site of *nosR*, which is located upstream of the operon (see Supplementary Fig. 7). The transcription start site was investigated under anaerobic conditions with NO_3_^-^ or N_2_O as the sole electron acceptor by 5′ rapid amplification of cDNA ends (5′ RACE).

**Figure 4:**
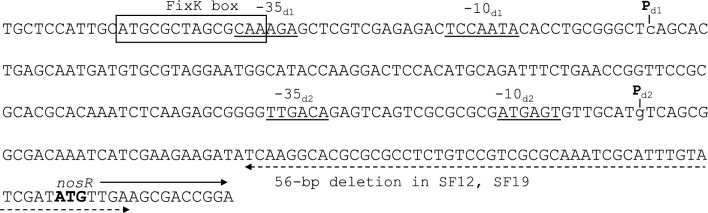
Transcriptional organization of *nosR* in *Bradyrhizobium ottawaense* SG09 and OO99^T^. P_d1_(c) is the transcription start site under N_2_O- and NO_3_^-^-respiring conditions. P_d2_(g) is the transcription start site under N_2_O-respiring conditions. -35/-10 consensus sequences preceding each transcription start site are indicated by underlining. Putative FixK box located upstream of P_d1_ is shown in the box. The translational start codon (ATG) of *nosR* is shown in bold case. The promoter sequences of *B. ottawaense* SG09 and OO99^T^ are completely identical. The dotted underlined region indicates the 56 bp deleted in strains SF12 and SF19 (see Supplementary Fig. 7 for details).

Under N_2_O-respiring conditions, two start sites were detected in *B. ottawaense* SG09 at C and G, 212 and 79 nucleotides (nt) upstream of the *nosR* start codon, respectively (P_d1_ and P_d2_, respectively; Fig. 4, Supplementary Fig. 8). The -35/-10 consensus sequence was predicted upstream of the two transcription start sites. In addition, the putative FixK box was predicted upstream of P_d1_. On the other hand, under NO_3_^-^-respiring conditions, a single transcription start site, P_d1_, was observed in* B*. *ottawaense* SG09 (Fig. 4 and Supplementary Fig. 8). We also confirmed that *B. ottawaense* OO99^T^ has identical promoter sequences and two transcription start sites (P_d1_ and P_d2_; Fig. 4 and Supplementary Fig. 8). In *B. ottawaense* OO99^T^, two transcription start points (P_d1_ and P_d2_) were detected under NO_3_^-^-respiring conditions, but comparing the band intensities, it was clear that P_d1_, which was also detected in SG09, was strongly transcribed. We additionally examined the transcription start site in USDA110^T^ under N_2_O-respiring conditions and detected a single site (G, 84 nucleotides upstream of *nosR*) that was identical to the previously reported site of USDA110^T^ under NO_3_^-^-respiring conditions^24,33^ (see Supplementary Fig. 8). Our results indicate that, unlike *B. diazoefficiens* USDA110^T^, the *nosR* of *B. ottawaense* has two transcription start sites under N_2_O-respiring conditions.

### Two *B. ottawaense* strains with low N_2_O-reducing activity

Among the *B. ottawaense* analyzed in this study, two strains SF12 and SF19 showed low N_2_O-reducing activity and *nosZ* expression (Fig. 1a, Table 1). When compared *nos* gene clusters (see Supplementary Fig. 7a, b), we could not identify the differences responsible for the N_2_O-reducing activity as their *nos* genes are identical in amino acid sequence. However, when compared the genomic sequence of the upstream region of *nosR*, 56 bp deletion was commonly observed in the low N_2_O-reducing activity strains SF12 and SF19; the deleted region includes the start codon (ATG) of *nosR* (Fig. 4 and Supplementary Fig. 7c). To confirm that 56 bp deletion is the cause of low activity, the deletion mutants of SG09 and OO99^T^(SG09Δ56, OO99^T^Δ56) were generated. In the 56 bp deletion mutants, both N_2_O-reducing activity and *nosZ* expression levels were decreased to levels comparable to those of SF12 and SF19 (Table 3), confirming that 56 bp deletion is the cause of the low activity of SF12 and SF19.

**Table 3:** Relative expression of *nosZ* and N_2_O-reducing activity in the 56 bp deletion mutant of *B. ottawaense.*

	Relative expression	N_2_O-reducing activity (nmol h^−1^ protein^−1^)
*B. ottawaense* SG09Δ56	24.0 ± 7.9	2.0 ± 0.3
*B. ottawaense* OO99^T^Δ56	38.4 ± 5.0	4.0 ± 0.3
*B. ottawaense* SG09*	211.5 ± 58	13.2 ± 1.6
*B. ottawaense* OO99^T^*	163.5 ± 81	11.8 ± 0.9
*B. diazoefficiens* USDA110^T^ *	1.0 ± 0.48	2.0 ± 0.4

*The wild-type data were obtained from Fig. 1a and Table 1.

## Discussion

In this study, we demonstrated that *B. ottawaense* has higher N_2_O-reducing activity than that of *B. diazoefficiens*. In a previous study, *B. diazoefficiens* mutants with high N_2_O-reducing activity (*nos*^++^ mutants) were generated, and the mutants mitigated N_2_O emission at the laboratory and field levels^11,34^. One of the *nos*^++^ mutants (5M09) was established as a non-genetically modified organism; however, this strain has 66 mutations in the genome, raising concerns for actual agricultural use. The *B. ottawaense* described in this study is a WT strain that exhibits high N_2_O reduction activity comparable to that of the artificially generated USDA110^T^ *nos*^++^ mutant strains; both the *nos*^++^ mutants (5M09) and *B. ottawaense* exhibited approximately fivefold higher N_2_O reduction activity than the WT USDA110^T^ (Fig. 1 and^25^). Furthermore, we demonstrated that SG09 inoculation resulted in almost no N_2_O release in the rhizosphere. Notably, negative N_2_O flux was observed under a 50 ppm N_2_O gas phase in the laboratory experiment, suggesting a system is in place to reduce high N_2_O concentrations (Fig. 2). Given that GHG reduction is a current key issue, *B. ottawaense* is quite beneficial as it can contribute to the mitigation of N_2_O in agricultural fields.

High N_2_O-reducing ability is considered adaptive in environments with high N_2_O concentrations. *B. ottawaense* was first isolated from a soybean field in Canada in 2012 as a novel species^29,35^. Other *B. ottawaense* isolates have been reported from soybean and peanut fields in China and Japan and woody legumes in Ethiopia^36–39^. *B. ottawaense* can form nodules in soybeans, but it is rarely detected in soybean fields in Japan^37^, suggesting that it is adapted to different environments than those of conventional soybean rhizobacteria such as *B. diazoefficiens*, *B. japonicum*, and *B. elkanii*. N_2_O reduction occurs preferentially over NO_3_^-^ reduction^40^, and *B. ottawaense* can grow better than *B. diazoefficiens* under N_2_O-respiring conditions (see Supplementary Fig. 3). Therefore, it is possible that the ability to reduce N_2_O, as in *B. ottawaense,* may have been important to survive in specific environments.

In the current study, the N_2_O-reducing activity of bradyrhizobia showed a similar behavior to the expression of the *nosZ* gene. *B*. *ottawaense* strains with high N_2_O-reducing activity (SG09 and OO99^T^) strongly express the *nosZ* gene under both N_2_O- and NO_3_^-^-respiring conditions (Figs. 1 and 3, Table 1). In addition, *Bradyrhizobium* with low N_2_O-reducing activity (USDA110^T^, SF12, SF19) showed relatively low *nosZ* expression compared to that of high N_2_O-reducing strains (Fig. 1, Table 1). Given the relatively high homology of *nosZ* between *B. diazoefficiens* and *B. ottawaense* (92% identity in amino acid sequence, see Supplementary Fig. 7a), our results suggest that the N_2_O-reducing activity of *Bradyrhizobium* is determined by the expression of the *nosZ* gene rather than NosZ protein activity. However, further experiments, such as swapping of promotor or coding regions of *nos*Z gene, are required to prove this idea.

To investigate the cause of high *nosZ* expression in *B. ottawaense*, we first focused on the NasST regulatory system and examined whether it is functional in *B. ottawaense*. In the *nasS* deletion mutants (OO99^T^Δ*nasS* and SG09Δ*nasS*), *nosZ* expression levels increased under N_2_O-respiring condition but not increased under NO_3_^-^-respiring condition (Table 1), indicating that the NasST regulatory system is functional in *B. ottawaense* as in *B. diazoefficiens*^25,33^. In addition, it seems that the NasST regulatory system is not a main factor for the high expression in *B. ottawaense* because the *nosZ* expression of WT *B. ottawaense* (211 in SG09, 163 in OO99^T^) was higher than that of USDA110 Δ*nasS* (3.4) under N_2_O-respiring conditions (Table 2).

Analysis of Δ*nasS* mutants also showed that *nosZ* gene expression levels are not directly reflected in N_2_O-reducing activity. *B. ottawaense* Δ*nasS* mutants exhibited higher *nosZ* gene expression than that of WT (Table 2), but N_2_O-reducing activity did not significantly differ between WT and Δ*nasS* mutants (see Supplementary Fig. 6). In addition, when comparing *nosZ* expression in Δ*nasS* mutants*, B. ottawaense* demonstrated a 100-fold higher expression than that of *B. diazoefficiens* (Table 2)*,* but N_2_O-reducing activity only slightly differed (see Supplementary Fig. 6). The lack of linearity between gene expression and N_2_O-reducing activity may indicate upper limits for NosZ protein activity. This may be due to translation efficiency or depletion of the components required for NosZ activity, such as copper and electrons, during N_2_O reduction. Moreover, NosZ protein activation requires highly complex pathways, such as sequential metal trafficking and assembly to copper sites via NosDFY^41^. The exact cause is presently unknown, but the aforementioned factors may define the upper limit of N_2_O reduction activity in bradyrhizobia.

Previous studies have shown the single or two transcription start sites of *nosR* in *B*. *diazoefficiens* USDA110^T^ under free-living (aerobic), NO_3_^-^-respiring (anaerobic), and symbiotic conditions^24,33,42^. In the current study, we present the first analysis of transcription start point under N_2_O-respiring condition. In *B*. *diazoefficiens* USDA110^T^, single transcription start point was observed regardless of different electron acceptors (N_2_O or NO_3_^-^). In contrast, *B*. *ottawaense* has a variable transcription start point depending on the electron acceptors: two transcription start points were detected under N_2_O respiration conditions in both SG09 and OO99^T^. Changes in the transcription start site depending on two different electron acceptor have been reported in studies on *Geobactor*^43^. Also, genome-wide analysis of transcription start sites in *Clostridium* identified several metabolism-related genes with multiple transcription start sites that change depending on the substrate^44^. Although the importance of having multiple transcription start sites has not been fully elucidated, it is considered an important regulatory mechanism of gene expression because it largely influences transcription efficiency, translation initiation, and protein abundance^45^. Changes in the transcription start sites of *B. ottawaense nosR* depend on the type of electron acceptor, which may be part of the *nos* genes expression regulatory mechanism in the denitrification system. Mutations in the transcription start sites will clarify their importance in the regulation of *nos* gene expression.

Genome sequence comparisons of high and low N_2_O-reducing activity strains revealed a novel determinant of activity. Incidentally, 56 bp deletion in the upstream region including translational start codon (ATG) of *nosR* was detected specifically in the low N_2_O-reducing activity strains, SF12 and SF19 (Fig. 4 and Supplementary Fig. 7), and introducing the deletion in the high N_2_O-reducing activity strains (SG09 and OO99^T^) reduced *nosZ* gene expression and N_2_O-reducing activity (Table 3). These results are consistent with previous studies where decreased *nosZ* expression was observed in artificially generated *nosR*-deleted *Pseudomonas aeruginosa* strains in which the *nos* genes were encoded in a single operon similar to that of *Bradyrhizobium*^46^. Since NosR functions as an electron donor for NosZ and regulates the transcription of *nos* genes^47^, the 56 bp deletion including the translational start codon may impair the function of NosR protein, causing a decrease in *nosZ* gene expression and N_2_O-reducing activity. As partial gene deletion is among the driving forces for environmental adaptation or functional evolution in bacteria^48–50^, the strains with natural deletion isolated in the present study may have evolved to adapt to environments with limited denitrification substrates. Accordingly, examining the distribution and abundance of high and low N_2_O-reducing activity strains in various environments may reveal the importance of N_2_O-reducing activity in environmental adaptation.

In summary, we demonstrated that the N_2_O-reducing activity of *B. ottawaense* is significantly higher than that of conventional strains, and this activity is achieved via high *nosZ* expression. Since N_2_O is a GHG mainly generated in agricultural lands, developing strategies for reducing N_2_O emissions from agricultural lands is an urgent task. The *B. ottawaense* we reported here has great potential for GHG mitigation in the rhizosphere owing to its high N_2_O-reducing activity. In addition, the regulatory mechanism of *nos* gene expression we elucidated in this study will be useful for developing and identifying bacteria with higher GHG-reducing ability. Further studies on the ecology of *B. ottawaense* including its compatibility with legume crops and competitiveness with other indigenous rhizobacteria are needed to improve its utility on actual agricultural land.

## Methods

### Bacterial strains, isolation, and genome analysis

The type strain *B*. *ottawaense* OO99^T^ was purchased from the Microbial Domain Biological Resource Centre HAMBI (Helsinki, Finland). The type strain *B. diazoefficiens* USDA110^T^ was provided by Dr. Michael J. Sadowsky at University of Minnesota. We used the culture stock of the *nosZ* deleted mutant *B. diazoefficiens* USDA110Δ*nosZ* generated by Hirayama et al. 2011^51^. The *B*. *ottawaense* strains used in this study are listed in Supplementary Table 1. Eight strains (SG09, SG10, SG20, SG23, TM102, TM233, and TM239) have been reported by Wasai-Hara et al.^17^, and the other strains were isolated by the same procedures. For whole genome sequencing, genomic DNA was extracted using a Bacteria GenomicPrep Mini Spin Kit (Cytiva, Tokyo, Japan). DNA libraries were prepared using a Nextera Sample Preparation Kit (Illumina, San Diego, CA, USA), and the 300-bp paired-end libraries were sequenced using Illumina Miseq (Illumina). Subsequently, 20 bp of the 5′ and 3′ ends were trimmed, and the genomes were assembled using CLC Genomics Workbench ver. 8.5.1 (Illumina). Genome annotation was performed using DFAST^52^.

### N_2_O-reducing activity

N_2_O-reducing activity was determined by culturing the bacteria under anaerobic conditions with 1% N_2_O supplemented as the sole electron acceptor. The N_2_O concentration was measured using a gas chromatograph (GC2014; Shimadzu, Kyoto, Japan) equipped with a thermal conductivity detector and Porapak Q column (GL Sciences, Tokyo, Japan). Bacterial strains were aerobically cultured for over 6 h in a 75-mL test tube with an air-permeable plug containing 10 mL HM liquid medium^53^ supplemented with 0.1% (w/v) arabinose and 0.025% (w/v) yeast extract at 28 °C with shaking at 200 rpm. Thereafter, the appropriate volume of bacterial culture was added to new tubes containing 10 mL HM medium to reach an optical density (OD) at 660 nm (OD_660_) of 0.05. The OD was measured using a test tube 25 mm diameter (TEST25NP; AGC Techno Glass Co., Ltd., Shizuoka, Japan). The test tube was closed with a butyl rubber cap, and the gas phase was replaced with 4.98% N_2_O + 95.02% N_2_ gas following overnight (12–14 h) culture to induce N_2_O reduction metabolism. Subsequently, the gas phase was again replaced with 100% N_2_ gas, after which 100% N_2_O was supplemented to adjust to a final concentration of 1%. Finally, the test tube was incubated at 28 ºC with shaking at 200 rpm, and 100 µL of gas phase was withdrawn every 1–3 h and subjected to the gas chromatography. To calculate the N_2_O-reducing activity per total protein, calibration curves for protein content at OD_660_ were developed for both *B. ottawaense* and *B*. *diazoefficiens*. The total protein content was calculated from the measured OD_660_. Protein content in the supernatants was measured using a protein assay kit (Bio-Rad Laboratories, Hercules, CA, USA).

### N_2_O flux experiment

N_2_O flux in the soybean rhizosphere was measured using a previously described method with modifications^11^ (see Supplementary Fig. 4). Bacterial strains (*B. japonicum* USDA6^T^, *B. diazoefficiens* USDA110^T^ and *B. ottawaense* SG09) were aerobically cultured in HM liquid medium at 30 °C for 1 week, after which the prepared bacterial suspension was adjusted to 1 × 10^8^ cells mL^−1^ using sterilized water. Soybean seeds (*Glycine max*, cv. Enrei (GmJMC025) seeds acquired from Genebank Project NARO, Japan) were sterilized using 0.5% sodium hypochlorite were sown in Leonardo Jar pots—at three seeds per pot—containing sterilized vermiculite and were inoculated with 1 mL of bacterial suspension. Four pots were prepared per each inoculated strain. The seeds were cultivated in a growth chamber at 25 °C for 16 h in light and 8 h in the dark. Thinning was performed on the third day after sowing, leaving an individual plant that was in the best germination state, and cultivation continued for another 33 days. A nitrogen-free hydroponic solution was periodically added to the pot during cultivation. After cultivation, plant shoots system were decapitated, and the root system was gently immersed in water to remove excess vermiculite. The root system was transferred to a 100-mL glass vial containing 30 mL soil obtained from the Kashimadai experimental field (38°27′36.0″N 141°05′24.0″E, at the permission of Tohoku University, Japan). The soil was previously sieved through a 2mm mesh sieve to remove large soil aggregates and stones. In addition, 5 ml of sterile distilled water was added to the glass vials. Thereafter, the vials with the roots, soil, and water were incubated aerobically at 25 °C for 20 days to induce nodule degradation: the vials were covered with soft wiping cloth to maintain aeration during the incubation period. After the incubation period, for determining the N_2_O flux, the vials were sealed with butyl-rubber caps and kept for 240–360 min in the following treatments: (1) atmospheric condition and (2) with the addition of 50 ppm of N_2_O, in four replicates. N_2_O concentration in the gas phase of vials was determined a gas chromatograph (GC2014; Shimadzu) equipped with a ^63^Ni electron capture detector and tandem Porapak Q columns (GL Sciences; 80/100 mesh; 3.0 mm × 1.0 m and 3.0 mm × 2.0 m).

### Expression analysis

*nosZ* gene expression levels were measured under N_2_O- and NO_3_^-^-respiring conditions. For N_2_O-respiring conditions, cells were prepared the same as described above. 3 h after exposure to 1% N_2_O conditions, a 1 mL phenol solution (10% phenol in ethanol) was added to the 1-mL culture to stop metabolism. After centrifugation, the pellets were stored at − 80 °C until further processing. For NO_3_^-^ respiring conditions, cells were anaerobically grown in 20 mL HM medium supplemented with 10 mM KNO_3_ in a 75-mL test tube. The OD_660_ was initially adjusted to 0.05 and monitored to induce the exponential growth phase of cells. When OD_660_ reached 0.1, the cells were collected as described above. Subsequently, total RNA was isolated using the hot-phenol method as described previously^54^, followed by DNase I treatment (RQ1; Promega, Madison, WI, USA) and further purification using RNA Clean & Concentrator-5 (Zymo Research, Irvine, CA, USA). First-strand cDNA was synthesized using 500 ng RNA as a template and SuperScript IV Reverse Transcriptase (Invitrogen, Waltham, MA, USA) according to the manufacturer’s instructions. RT-qPCR was performed using a LightCycler Nano Instrument (Roche, Basel, Switzerland), LightCycler® FastStart DNA Master^PLUS^ SYBR® Green I (Roche), and specific primers for *sigA* (sigAf/sigAr) and *nosZ* (nosZ_qPCR_F/ nosZ_qPCR_R) (see Supplementary Table 4) at an annealing temperature of 60 ºC for 50 cycles. Relative expression calculated using the 2^−ΔΔCt^ method^55^ was normalized to *sigA* expression.

### NosZ activity staining

*B. ottawaense* SG09 and *B. diazoefficiens* USDA110^T^ were cultured overnight under N_2_O-respiring conditions. After centrifugation of the cultured cells, total protein was extracted using lysis buffer (CelLytic B; Sigma-Aldrich, St. Louis, MO, USA) and sonication (BIORUPTOR; BM Equipment Co., Ltd., Tokyo, Japan). Protein content in the supernatants was measured using a DC-protein assay (Bio-Rad). For DOC-PAGE, approximately 9.6 µg of total protein was used as the non-diluted sample (1×, Fig. 3). After electrophoresis, each gel was immersed in the buffer containing 25 mM Tris, 192 mM glycine, and 1 mM methyl viologen (pH 8.3). Subsequently, Ti(III)-citrate was used to reduce methyl viologen, and N_2_O-saturated H_2_O was added for the in-gel N_2_O-reducing enzymatic reaction. Band signal intensity was determined using an Image J macro, Band/Peak Quantification^56^. All experiments except for protein quantification were performed under an N_2_ atmosphere. CBB staining was performed after N_2_O-reducing activity staining to confirm protein content.

### *nasS*, *nosZ*, and 56-bp deletion mutants

*nasS* deletion mutants were generated using the in-frame markerless method. The deletion region was determined as in the *B. diazoefficiens* USDA110^T^ *nasS* mutant strain (5M09) reported by Sánchez et al.^25^. Briefly, pK18mobsacB-Ω was created by replacing the kanamycin resistance gene coding region of the suicide vector pK18mobsacB^57^ with a streptomycin-spectinomycin resistance gene (*aadA*). pK18mobsacB was digested with NcoI and BglII to obtain 5.1 kb of linear DNA with 0.6 kb of the kanamycin resistance gene partially deleted. The aadA fragment to be introduced was amplified by PCR using primers aadA_F_IF and aadA_R_IF and Prime STAR® Max DNA Polymerase (Takara Bio Inc., Shiga, Japan) (details on the primers are shown in Supplementary Table 4) with pHP45Ω^58^ as a template. Thereafter, 1.1 kb of amplified DNA was extracted using Wizard® SV Gel and a PCR Clean-Up System (Promega). The resulting linear pK18mobsacB and aadA were combined using an In-fusion HD cloning kit (Takara Bio Inc.) and transformed *E. coli* DH5α according to the manufacturer’s instructions.

The up- and downstream regions of the *nasS* gene were amplified by PCR using primers Bo_nasSdel_F1/R1 and Bo_nasSdel_F2/R2 (see Supplementary Table 4 for details on primers) and Prime STAR® Max DNA Polymerase (Takara Bio Inc.). Amplified fragments were combined by overlap extension PCR and inserted into the SmaI site of pK18mobsacB-Ω using an In-fusion HD Cloning Kit (Takara Bio Inc.). The sequence of the introduced fragment was confirmed by sequencing, and the resultant plasmid was designated pMS187. Transmission of pMS187 to *B. ottawaense* strains and homologous recombination of the *nasS* region were performed by triparental mating with a mobilizing *E. coli* HB101 strain harboring the pRK2013^59^ helper plasmid. Next, *B. ottawaense* SG09 or OO99^T^, *E. coli* DH5α harboring pMS187, and *E. coli* HB101 harboring pRK2013 were mixed and cultured for mating. Transconjugants were selected by resistance to streptomycin (Sp, 100 µg/mL), spectinomycin (Sm, 100 µg/mL), and polymyxin (Px, 50 µg/mL) and sensitivity to sucrose (10%). The single crossover strains were further cultured in HM medium without antibiotics, and deletion mutants that showed Sp/Sm sensitivity and sucrose resistance—SG09Δ*nasS* and OO99^T^Δ*nasS*—were obtained.

Δ*nosZ* and 56 bp deletion mutants were generated using the same methods as for the *nasS* mutants with the pK18mobsacB-Ω vector. Briefly, the up- and downstream regions of the *nosZ* gene were amplified by PCR using primers SG09_nos-1F/1R and SG09_nos-2F/2R (see Supplementary Table 4 for details on the primers) and Prime STAR® max DNA Polymerase (Takara Bio Inc.). The amplified fragments were combined by overlap extension PCR using primers SG09_nos-1F/1R and SG09_nos-2F/2R (see Supplementary Table 4) and Prime STAR® max DNA Polymerase (Takara Bio Inc.). The PCR fragments and pK18mobsacB-Ω were digested with EcoRI and HindIII and then ligated using a DNA Ligation Kit (< Mighty Mix > ; Takara Bio Inc.). Thereafter, triparental mating was performed using the sequence-introduced vector as described above.

For 56-bp deletion mutants, the up- and downstream regions of the 56-bp region were amplified by PCR using primers 56del_F1/R1 and 56del_F2/R2 (see Supplementary Table 4) and then combined and inserted into the SmaI site of pK18mobsacB-Ω using an In-fusion HD Cloning Kit (Takara Bio Inc.). Thereafter, triparental mating was performed using the sequence-introduced vector as described above. The generated 56 bp deletion mutants were designated SG09Δ56 and OO99^T^Δ56.

### 5′ RACE

5′ RACE experiments were performed using a 5′/3′ RACE kit, 2^nd^ Generation (Roche). Briefly, the total RNA of *B. ottawaense* and *B*. *diazoefficiens* strains were isolated from cells grown under N_2_O- and NO_3_^-^- respiring conditions using the hot-phenol method as described above. cDNA synthesis and amplification of the 5′- region of *nosR* were conducted according to the manufacturer’s instructions using the primers listed in Supplementary Table 4 (Bw_SP1, SP2, and SP3 for *B*. *ottawaense* strains, R_SP1, SP2, and SP3 for *B. diazoefficiens* strains). The amplified fragments were sequenced to determine the transcription start site.

### Statistical analysis

Differences in N_2_O reducing activities between all strains tested were evaluated using Tukey’s test after ANOVA analysis. Differences in N_2_O flux and *nosZ* gene expression between the two strains were evaluated using Student’s* t* tests at a significance level of 0.05.

### Supplementary Information

Supplementary Information.

## Data Availability

Genome data are available in NCBI (https://www.ncbi.nlm.nih.gov/), and accession numbers are detailed in the Supplementary Information files.
