# Supplementary material for: Bradyrhizobium ottawaense efficiently reduces nitrous oxide through high nosZ gene expression
Source: Sci Rep. 2023 Nov 1;13:18862. doi: 10.1038/s41598-023-46019-w (PMC10620151; doi:10.1038/s41598-023-46019-w)
Supplement: Supplementary file 1 — Supplementary Information. [file 41598_2023_46019_MOESM1_ESM.pdf]

***Bradyrhizobium ottawaense*** **efficiently reduces nitrous oxide through high** ***nosZ*** **gene expression**

## Supplementary information

Sawa Wasai-Hara[1, 2], Manabu Itakura[2], Arthur Fernandes Siqueira[2], Daisaku Takemoto[1], Masayuki Sugawara[2], Hisayuki Mitsui[2], Shusei Sato[2] , Noritoshi Inagaki[1], Toshimasa Yamazaki[1], Haruko Imaizumi-Anraku[1], Yoshikazu Shimoda[1*], and Kiwamu Minamisawa[2*]

[1]National Agriculture and Food Research Organization (NARO), Tsukuba, Ibaraki 305-8604, Japan
[2]Graduate School of Life Sciences, Tohoku University, Katahira, Aoba-ku, Sendai 980-8577, Japan

*Corresponding author: Y.S., yshimoda@affrc.go.jp, K.M., kiwamu.minamisawa.e6@tohoku.ac.jp

## Methods

***Phylogenic analysis* (Supplementary Fig. 1)**

Thirty-one genes belong to AMPHORA were extracted from the information annotated by DFAST, combined, and aligned. A phylogenetic tree was generated using MEGAX (ver 10.2.6; https://www.megasoftware.net/, [1]) using the neighbor-joining method.

**Measurement of $N_2O$ reduction activity (Supplementary Fig. 2)**

The $N_2O$ reduction activity was performed as described in the main text and the $N_2O$ reduction speed was normalized by optical density (OD) at 660 nm ($OD_{660}$); $OD_{660}$ was measured in test tubes and calibrated to a value per 1-mL cuvette from a calibration curve.

***Growth experiment* (Supplementary Fig. 3)**

*B. ottawaense* SG09 and OO99$^T$ and *B. diazoefficiens* USDA110$^T$ were cultured under $N_2O$-respiring conditions. The strains were cultured HM liquid medium and initial $N_2O$ concentration was adjusted to 30%.

***Comparison of gene cluster* (Supplementary Fig. 5)**

Comparison of gene cluster organization and homology was performed using GenomeMatcher (ver 3.04, [2]).

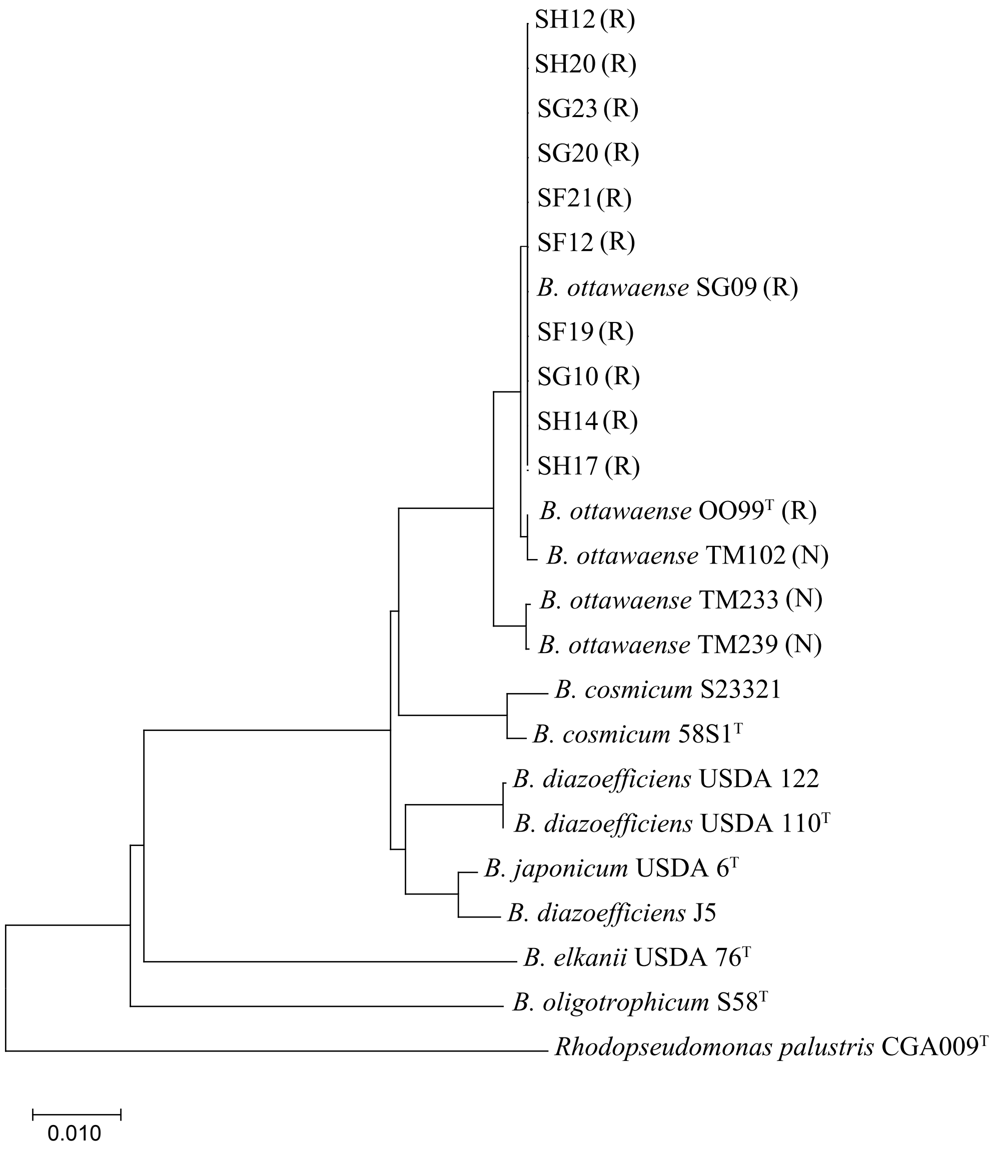


**Supplementary Fig. 1**. Phylogenic tree based on AMPHORA genes. Parentheses after the strain name indicate nodule-forming ability. R = nodule-forming strain (rhizobia), N = non-nodulation and non-diazotroph.

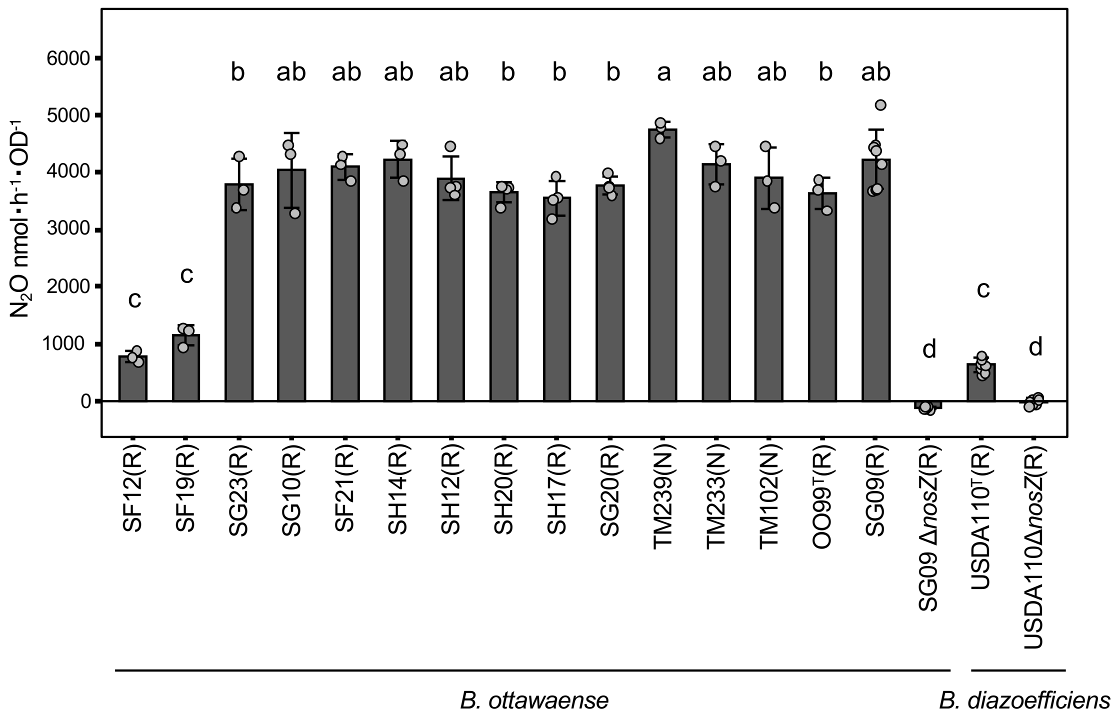


**Supplementary Fig. 2**. $N_2O$-reducing activity of *B. ottawaense* isolates, type strain $OO99^T$, *B. diazoefficiens* stain $USDA110^T$, and the *nosZ*-deficient strain (Δ*nosZ*) normalized by the value of $OD_{660}$. Different letters above the bars represent significant differences between inoculation treatments analyzed using Tukey's test after analysis of variance (ANOVA; $p < 0.05$). Parentheses after the strain name indicate nodule-forming ability. R = nodule forming strain (rhizobia), N = non-nodulation and non-diazotroph.

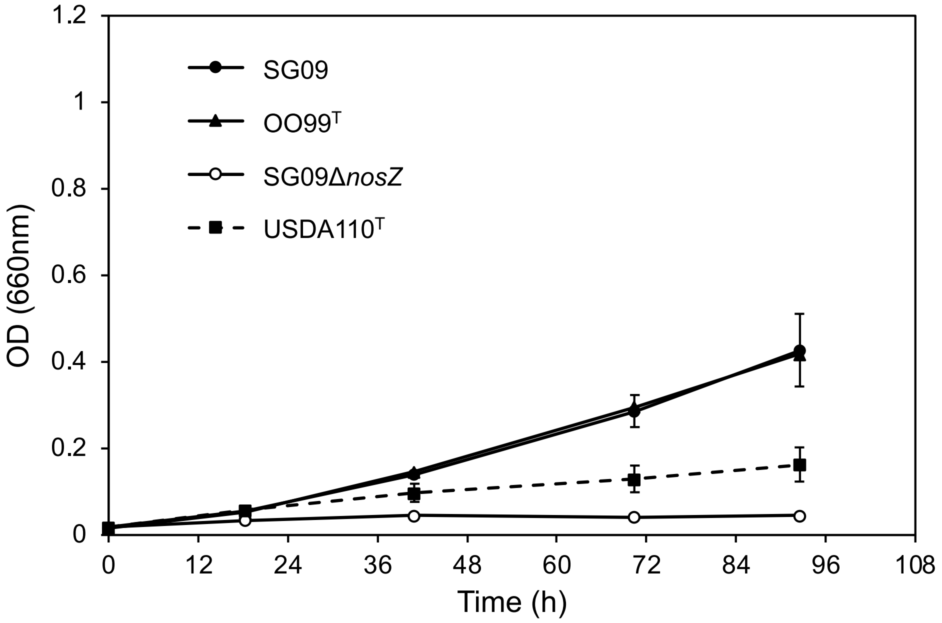


**Supplementary Fig. 3**. Growth curve of *Bradyrhizobium ottawaense* SG09, OO99$^T$, SG09 Δ *nosZ*, and *B. diazoefficiens* USDA110$^T$ under $N_2O$-respiring conditions. Error bars denote standard deviation ($n$ = 3).

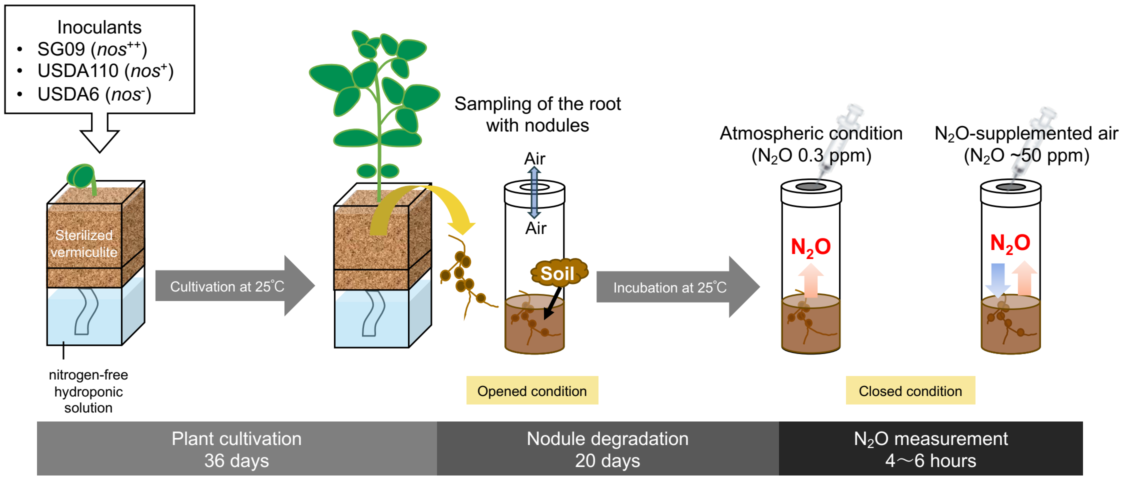


**Supplementary Fig. 4**

Overview of $N_2O$ flux experiment in the nodule decomposed soybean rhizosphere shown in Fig. 2. Four pots were prepared per each strain, and each strain was inoculated 1 mL cell suspension of 1 x $10^8$ cells/mL per pot. Plant cultivation was conducted in a growth chamber at 25°C with 18 hours of light and 6 hours of dark. $N_2O$ flux was determined by measuring the change in $N_2O$ concentration in the vials with sealing for 4-6 hours by using gas chromatograph.

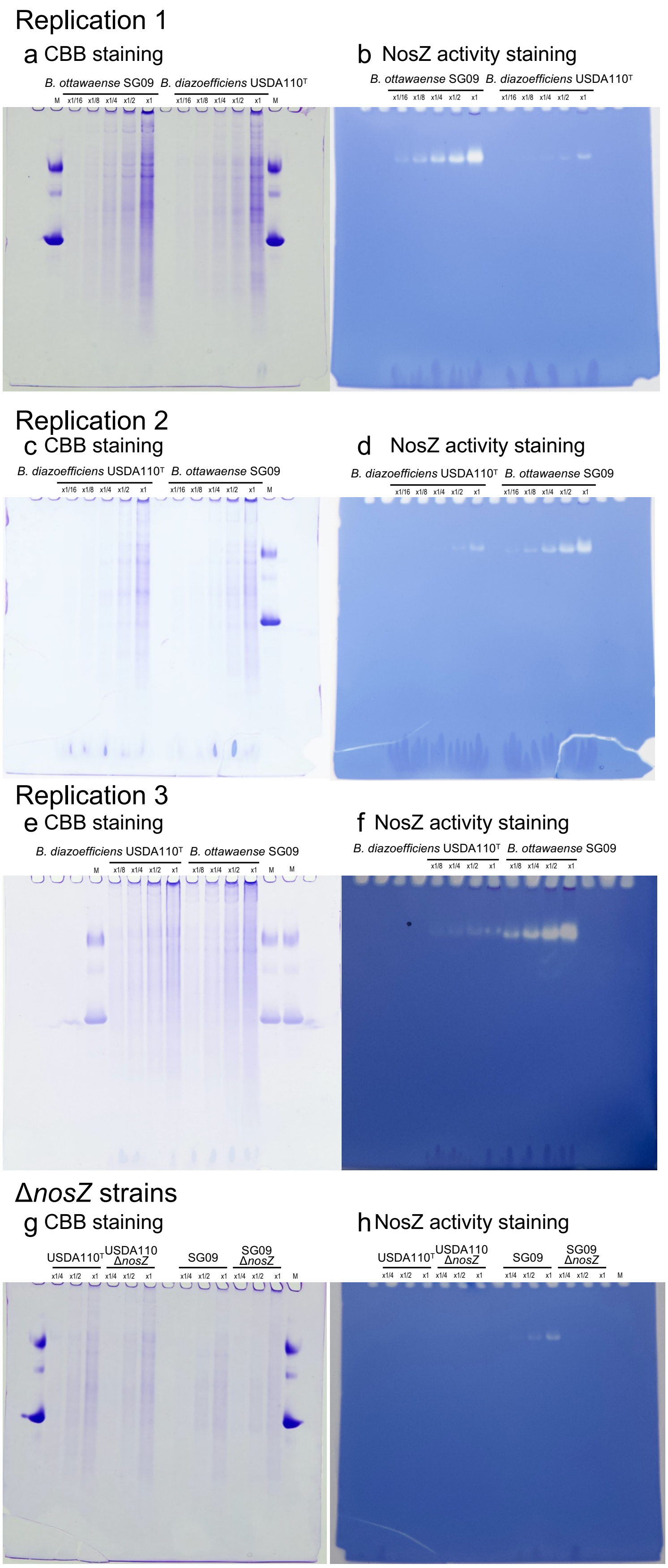
Replication 1
a CBB staining
B. ottawaense SG09
B. diazoefficiens USDA110T
M x1/16 x1/8 x1/4 x1/2 x1 x1/16 x1/8 x1/4 x1/2 x1 M
b NosZ activity staining
B. ottawaense SG09
B. diazoefficiens USDA110T
x1/16 x1/8 x1/4 x1/2 x1 x1/16 x1/8 x1/4 x1/2 x1
Replication 2
c CBB staining
B. diazoefficiens USDA110T
B. ottawaense SG09
x1/16 x1/8 x1/4 x1/2 x1 x1/16 x1/8 x1/4 x1/2 x1 M
d NosZ activity staining
B. diazoefficiens USDA110T
B. ottawaense SG09
x1/16 x1/8 x1/4 x1/2 x1 x1/16 x1/8 x1/4 x1/2 x1
Replication 3
e CBB staining
B. diazoefficiens USDA110T
B. ottawaense SG09
M x1/8 x1/4 x1/2 x1 x1/8 x1/4 x1/2 x1 M M
f NosZ activity staining
B. diazoefficiens USDA110T
B. ottawaense SG09
x1/8 x1/4 x1/2 x1 x1/8 x1/4 x1/2 x1
ΔnosZ strains
g CBB staining
USDA110T
USDA110 ΔnosZ
SG09
SG09 ΔnosZ
x1/4 x1/2 x1 x1/4 x1/2 x1 x1/4 x1/2 x1 x1/4 x1/2 x1 M
h NosZ activity staining
USDA110T
USDA110 ΔnosZ
SG09
SG09 ΔnosZ
x1/4 x1/2 x1 x1/4 x1/2 x1 x1/4 x1/2 x1 x1/4 x1/2 x1 M

**Supplementary Fig. 5**. Activity of the NosZ protein of *B. ottawaense* and *B. diazoefficiens* in replicate experiments. Coomassie brilliant blue staining (**a, c, e, g**) and NosZ-specific activity staining (DOC-PAGE, **b, d, f, h**) protein extracted from *B. ottawaense* SG09 and *B. diazoefficiens* USDA110$^T$. **a** and **b** are the full size gel images of Fig. 3. **g** and **h** include the Δ*nosZ* mutant of SG09 and USDA110. The numbers in each lane indicate the concentration (*x*) rate of extracted protein samples. 'M' indicates the protein size marker (60, 120, and 240 kDa were indicated).

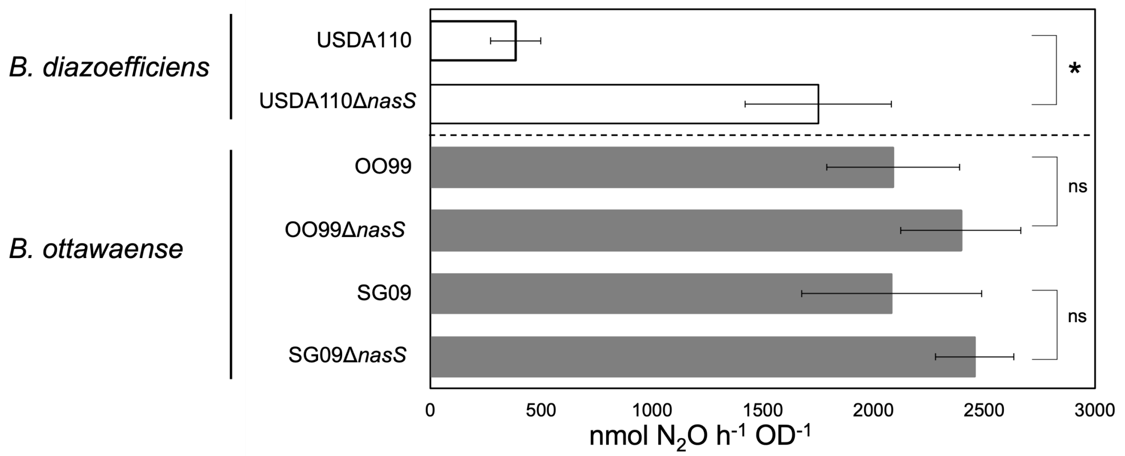


**Supplementary Fig. 6**. $N_2O$-reducing activity of the Δ*nasS* mutants of *B. ottawaense* SG09, OO99$^T$, and *B. diazoefficiens* USDA110$^T$. Asterisk represents significant difference at $p < 0.05$, $n = 4$, by *t*-test. ns = not significant.

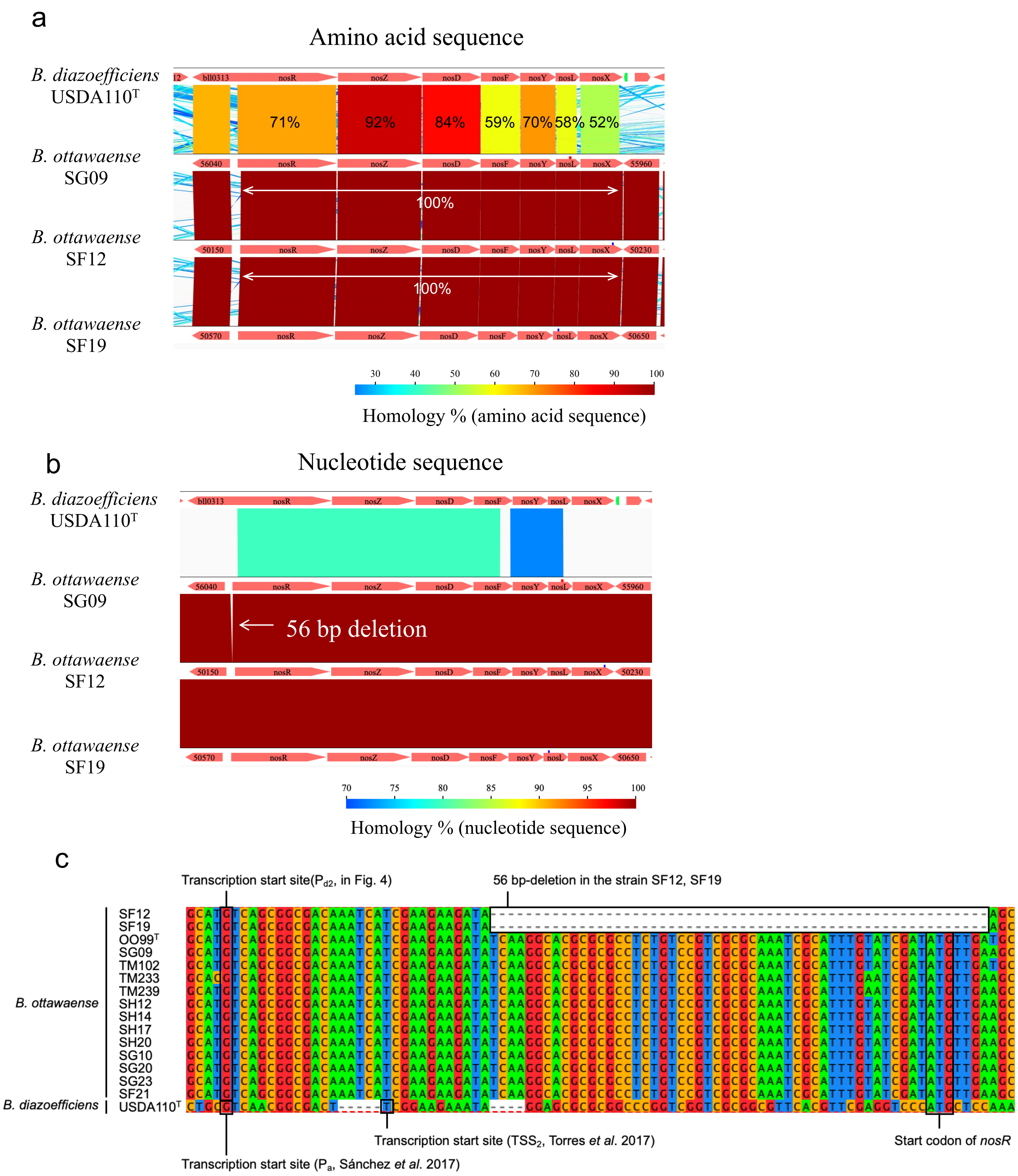


**Supplementary Fig. 7**. Homology of *nos* gene cluster amino acid (**a**) and nucleotide sequences (**b**). The pink arrow indicates gene configuration, and the colors among these genes indicate the homology between the upper and lower strains. Nucleotide sequence upstream of *nosR* and the 56 bp deletion site (**c**).

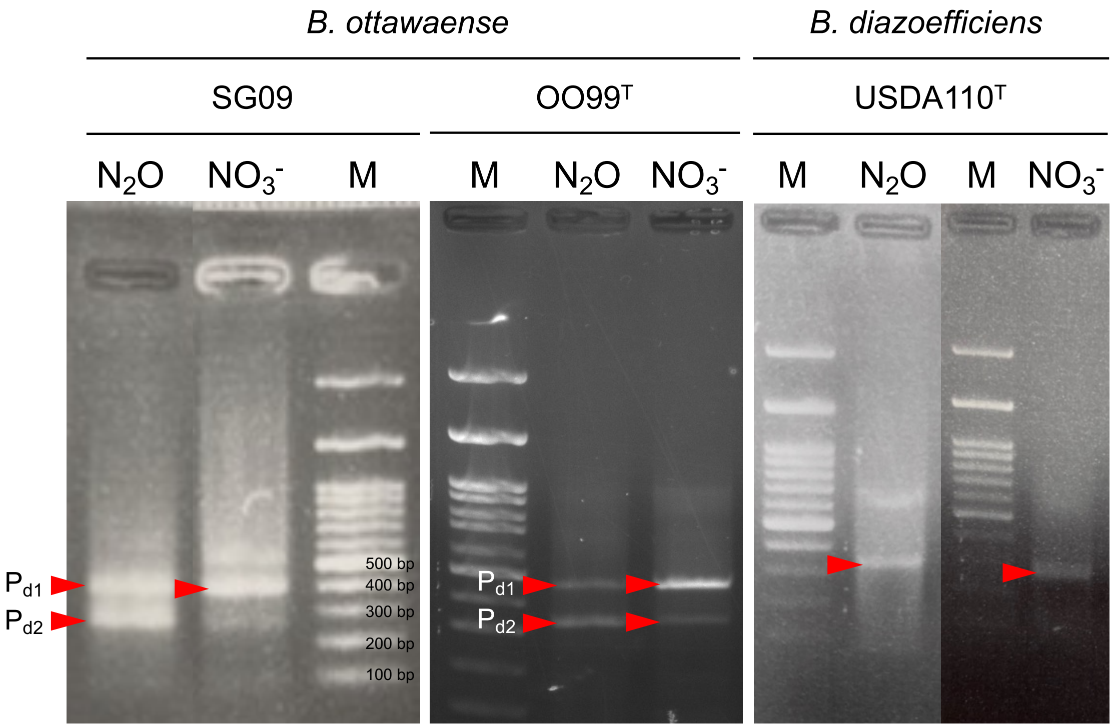


**Supplementary Fig. 8**. Electrophoresis images of 5′ RACE analysis in *Bradyrhizobium ottawaense* SG09, OO99$^T$, and *B. diazoefficiens* USDA110$^T$. The "$N_2O$" and "$NO_3$" lanes indicate samples obtained under $N_2O$- and $NO_3$-reducing conditions. Lane M is a size maker (FastGene 100 bp DNA Marker; Nippon Genetics, Tokyo, Japan). Red arrows indicate bands for which the sequence was determined.

**Supplementary Table 1**. Bacterial strains used in this study.

| Strain | Accession no. | Reference for genome sequence |
|---|---|---|
| USDA110$^T$(R) | BA000040.2 | [3] |
| OO99$^T$(R) | CP029425.1 | [4] |
| SG09(R) | AP021854.1 | [5] |
| TM102(N) | AP021855.1 | [5] |
| TM233(N) | BTIG01000001-BTIG01000413 | [5] |
| TM239(N) | BTIR01000001-BTIR01000077 | [5] |
| SG10(R) | BTIQ01000001-BTIQ01000335 | This study |
| SG20(R) | BTIH01000001-BTIH01000340 | This study |
| SG23(R) | BTII01000001-BTII01000335 | This study |
| SF12(R) | BTIN01000001-BTIN01000328 | This study |
| SF19(R) | BTIO01000001-BTIO01000828 | This study |
| SF21(R) | BTIP01000001-BTIP01000318 | This study |
| SH12(R) | BTIJ01000001-BTIJ01000507 | This study |
| SH14(R) | BTIK01000001-BTIK01000358 | This study |
| SH17(R) | BTIL01000001-BTIL01000343 | This study |
| SH20(R) | BTIM01000001-BTIM01000619 | This study |

Parentheses after the strain name indicate nodule-forming ability. R = nodule forming strain (rhizobia), N = non-nodulation and non-diazotroph.

**Supplementary Table 2**. Average nucleotide identity between *Bradyrhizobium ottawaense* isolates and type strain OO99$^T$

| | OO99$^T$(R) | SF12(R) | SF19(R) | SF21(R) | SG09(R) | SG10(R) | SG20(R) | SG23(R) | SH12(R) | SH14(R) | SH17(R) | SH20(R) | TM102(N) | TM233(N) | TM239(N) |
|---|---|---|---|---|---|---|---|---|---|---|---|---|---|---|---|
| OO99$^T$(R) | | 99.04 | 99.01 | 99.07 | 99.08 | 99.05 | 99.07 | 99.07 | 99.07 | 99.05 | 99.05 | 99.07 | 98.99 | 95.30 | 95.29 |
| SF12(R) | 99.04 | | 99.95 | 99.93 | 99.92 | 99.99 | 99.94 | 99.95 | 99.94 | 99.93 | 99.92 | 99.94 | 99.15 | 95.33 | 95.27 |
| SF19(R) | 99.01 | 99.95 | | 99.92 | 99.90 | 99.96 | 99.93 | 99.92 | 99.91 | 99.91 | 99.90 | 99.89 | 99.13 | 95.35 | 95.26 |
| SF21(R) | 99.07 | 99.93 | 99.92 | | 99.96 | 99.94 | 99.96 | 99.96 | 99.96 | 99.96 | 99.97 | 99.95 | 99.09 | 95.36 | 95.25 |
| SG09(R) | 99.08 | 99.92 | 99.90 | 99.96 | | 99.92 | 99.94 | 99.94 | 99.94 | 99.95 | 99.95 | 99.93 | 99.10 | 95.35 | 95.27 |
| SG10(R) | 99.05 | 99.99 | 99.96 | 99.94 | 99.92 | | 99.95 | 99.95 | 99.94 | 99.94 | 99.93 | 99.94 | 99.14 | 95.37 | 95.28 |
| SG20(R) | 99.07 | 99.94 | 99.93 | 99.96 | 99.94 | 99.95 | | 99.98 | 99.97 | 99.95 | 99.96 | 99.96 | 99.12 | 95.43 | 95.30 |
| SG23(R) | 99.07 | 99.95 | 99.92 | 99.96 | 99.94 | 99.95 | 99.98 | | 99.99 | 99.95 | 99.95 | 99.99 | 99.11 | 95.45 | 95.30 |
| SH12(R) | 99.07 | 99.94 | 99.91 | 99.96 | 99.94 | 99.94 | 99.97 | 99.99 | | 99.95 | 99.96 | 99.98 | 99.10 | 95.45 | 95.29 |
| SH14(R) | 99.05 | 99.93 | 99.91 | 99.96 | 99.95 | 99.94 | 99.95 | 99.95 | 99.95 | | 99.99 | 99.94 | 99.08 | 95.34 | 95.25 |
| SH17(R) | 99.05 | 99.92 | 99.90 | 99.97 | 99.95 | 99.93 | 99.96 | 99.95 | 99.96 | 99.99 | | 99.95 | 99.10 | 95.35 | 95.29 |
| SH20(R) | 99.07 | 99.94 | 99.89 | 99.95 | 99.93 | 99.94 | 99.96 | 99.99 | 99.98 | 99.94 | 99.95 | | 99.10 | 95.47 | 95.30 |
| TM102(N) | 98.99 | 99.15 | 99.13 | 99.09 | 99.10 | 99.14 | 99.12 | 99.11 | 99.10 | 99.08 | 99.10 | 99.10 | | 95.34 | 95.30 |
| TM233(N) | 95.30 | 95.33 | 95.35 | 95.36 | 95.35 | 95.37 | 95.43 | 95.45 | 95.45 | 95.34 | 95.35 | 95.47 | 95.34 | | 98.90 |
| TM239(N) | 95.29 | 95.27 | 95.26 | 95.25 | 95.27 | 95.28 | 95.30 | 95.30 | 95.29 | 95.25 | 95.29 | 95.30 | 95.30 | 98.90 | |

**Supplementary Table 3**. Homology of nos-regulating genes between *Bradyrhizobium ottawaense* SG09 and *B. diazoefficiens* USDA110$^{T}$

| | FixL | FixJ | $FixK_2$ | FixK box | NasS | NasT (ANTAR region) | *nosR* upstream* | RegS | RegR |
|---|---|---|---|---|---|---|---|---|---|
| nucleotide | 88% | 92% | 92% | SG09 **ATG**CGCTAGCG**CAA**<br>USDA110:**TTG**ATCCAGCG**CAA** | 88% | 96% (96%) | 48% | 93% | 93% |
| amino acid | 91% | 96% | 93% | - | 90% | 99% (100%) | - | 97% | 99% |

*The sequence between the *nosR* gene and the upstream hypothetical gene (reverse oriented) was compared (see Supplementary Fig. 5).

Homologies of the NasT-ANTR region [8] are shown in parentheses.

Supplementary Table 4. Primers used in this study.

| Primer mane | purpose | sequence (5′→3′) | Reference |
|---|---|---|---|
| nosZ_qPCR_F | qPCR | ACCCGCGAATTCCTCAAGAA | This study |
| nosZ_qPCR_R | qPCR | CTGTTGGCCTTGTCGTTCATG | This study |
| sigAf | qPCR | GAGAACCAGATGTCGCTTGC | [6] |
| sigAr | qPCR | TGGATGTCCTGCTCCTGAAG | [6] |
| Bo_nasSdel_F1 | nasS deletion | TCGAGCTCGGTACCCCGGAATCCACCAATGCCTTG | This study |
| Bo_nasSdel_R1 | nasS deletion | GCAGCTCCGAATCAGGCCTTGTTGGACCAGGACACCTCG | This study |
| Bo_nasSdel_F2 | nasS deletion | CGAGGTGTCCTGGTCCAACAAGGCCTGATTCGGAGCTGC | This study |
| Bo_nasSdel_R2 | nasS deletion | CTCTAGAGGATCCCCGGTCCTGGATACCCTCGATC | This study |
| aadA_F_IF | nasS deletion | CAGGGGATCAAGATCGTGGACATAAGCCTGTTCGG | This study |
| aadA_R_IF | nasS deletion | GCAGGCATCGCCATGAGTGCATCTAACGCTTGAGT | This study |
| 56del_F1 | 56 bp deletion | TCGAGCTCGGTACCCGTCGAGGTAGCCGTCGACCA | This study |
| 56del_R1 | 56 bp deletion | GGTATCGTCATCCGGTCGCTTATCTTCTTCGATGATTTGTCGCCG | This study |
| 56del_F2 | 56 bp deletion | CGGCGACAAATCATCGAAGAAGATAAGCGACCGGATGACGATACC | This study |
| 56del_R2 | 56 bp deletion | CTCTAGAGGATCCCCCGGCTTCAGGCTGTCCTTCA | This study |
| SG09_nos-1F | nosZ deletion | CTCGAATTCCGCTGATCTTCATTCTGTGG | This study |
| SG09_nos-1R | nosZ deletion | CAGATCGTCCCCGGGCGCTCGAGAAGAACACGTAG | This study |
| SG09_nos-2F | nosZ deletion | TCTCGAGCGCCCGGGGACGATCTGTTCGACGACAA | This study |
| SG09_nos-2R | nosZ deletion | CTCAAGCTTCGGAAACATGCGTAAGAGAA | This study |
| Bw_SP1 | 5' RACE | GAGGAAGATGAATCCCTGGAGCTG | This study |
| Bw_SP2 | 5' RACE | GCGGTACACTGGTACGATGG | This study |
| Bw_SP3 | 5' RACE | GGCGTAATCTTCGGCAGGTAT | This study |
| R_SP1 | 5' RACE | CAGGTACACGAAGCCCTGCAATTG | [7] |
| R_SP2 | 5' RACE | GCGATAGGCCGGTATGATAG | [7] |
| R_SP3 | 5' RACE | GGGCAATCTTCGACAGGTAA | [7] |

**References**


1 Stecher, G., Tamura, K. & Kumar, S. Molecular evolutionary genetics analysis (MEGA) for macOS. *Mol. Biol. Evol.* 37, 1237-1239 (2020).

2 Ohtsubo, Y., Ikeda-Ohtsubo, W, Nagata, Y. & Tsuda, M. GenomeMatcher: A graphical user interface for DNA sequence comparison. *BMC Bioinformatics*. **9**, 376 (2008).

3 Kaneko *et al*. Complete genomic sequence of nitrogen-fixing symbiotic bacterium *Bradyrhizobium japonicum* USDA110. *DNA Res*. **31**; 189-197 (2002).

4 Nguyen, H.D.T., Cloutier, S. & Bromfield, E.S.P. Complete Genome Sequence of *Bradyrhizobium ottawaense* OO99$^T$, an Efficient Nitrogen-Fixing Symbiont of Soybean. *Microbiol. Resour. Announc.* **7**, e01477-18 (2018).

5 Wasai-Hara, S. *et al*. Diversity of *Bradyrhizobium* in non-leguminous sorghum plants: *B. ottawaense* isolates unique in genes for $N_2O$ reductase and lack of the type VI secretion system. *Microbes Environ*. 35, ME19102 (2020).

6 Itakura, M. *et al*. Mitigation of nitrous oxide emissions from soils by *Bradyrhizobium japonicum* inoculation, *Nature Clim*. *Change* **3**, 208–212 (2013).

7 Sánchez, C., Mitsui, H. & Minamisawa, K. Regulation of nitrous oxide reductase genes by NasT-mediated transcription antitermination in *Bradyrhizobium diazoefficiens*. *Environ Microbiol Rep*. **9**, 389-396 (2017).

8 Shu, C.J. & Zhulin, I.B. ANTAR: an RNA-binding domain in transcription antitermination regulatory proteins. *Trends Biochem Sci*. **27**, 3-5 (2002).
